# Shape optimized acoustic metagratings for anomalous refraction under strong thermoviscous effects

**DOI:** 10.1038/s41598-024-76240-0

**Published:** 2024-11-03

**Authors:** Anton Melnikov, Sören Köble, Severin Schweiger, Steffen Marburg, David A. Powell

**Affiliations:** 1 Fraunhofer Institute for Photonic Microsystems IPMS, Dresden, Germany; 2 Chair of Vibro-Acoustics of Vehicles and Machines, Technical University of Munich, Munich, Germany; 3 School of Engineering and Technology, University of New South Wales, Canberra, Australia

**Keywords:** Acoustics, Fluid dynamics, Engineering

## Abstract

The recent development of microacoustic metagratings opens up promising possibilities for manipulating acoustic wavefronts passively, particularly in applications such as flat acoustic lenses and ultra-high frequency ultrasound imaging. The emergence of two-photon polymerization has made it feasible to precisely manufacture microscopic structures, as required when metagratings are scaled to MHz frequencies in airborne ultrasound. Nevertheless, the downsizing process presents another hurdle as the increased thermoviscous effects result in substantial losses that must be considered during the design phase. In this study, we propose two designs for microacoustic metagratings that refract a normally incident wave towards –35 ° at 2 MHz, consisting of single-body and two-body meta-atoms. The designs are created by employing shape optimization techniques that incorporate the linearized Navier–Stokes equations in every iteration starting from a neutral geometry. This ensures that the evolution of geometric key features responsible for anomalous refraction fully accounts for thermoviscous effects, as would be the case during evolution in nature where the full set of physics is always active. Subsequently, we experimentally evaluate the effectiveness of these metagratings by employing a capacitive micromachined ultrasonic transducer as the sound source and an optical microphone as the detector, covering a frequency range from 1.8 to 2.2 MHz. Our findings confirm the single-body geometry reported in the literature and show an alternative geometry for two-body design, showcasing the successful utilization of two-photon polymerization for manufacturing microscopic acoustic metamaterials.

## Introduction

Acoustic metamaterials have revolutionized the design of acoustic materials with unique properties^1–4^. Consequently, a wide range of applications have emerged, including acoustic cloaking^5–7^, subwavelength acoustic barriers^8–10^, flat acoustic lenses^11–13^, particle manipulation^14^, and ultrasonic imaging with subwavelength resolution^15–17^. Experimental studies have demonstrated the effectiveness of gradient metasurfaces in wavefront control, both in reflecting^18,19^ and refracting^20–26^ designs. Notably, gradient acoustic metasurfaces have been successfully employed in acoustic Fresnel lenses^18,21,23,24,26,27^. However, the resolution of such devices is limited by the wavelength, necessitating the use of high or even ultra-high frequency ultrasound for applications requiring finer resolution. As the frequency increases, especially in the airborne ultrasound domain, the thermoviscous layers at sound-hard boundaries introduce more significant losses^28,29^. Gradient acoustic metasurfaces, employing thin acoustic channels and often incorporating Helmholtz resonators, are susceptible to severe thermoviscous effects that can interfere with their functionality. To address this limitation, among other things, acoustic metagratings have emerged as a promising alternative for the ultra-high frequency range^30–37^. The larger geometrical features of metagratings, approaching the wavelength scale, make them more suitable for scaling in comparison to gradient metasurfaces^38^. Reference^38^ demonstrates that the thermoviscous effects (e.g. linearized Navier–Stokes equations) nevertheless must be accounted for when an acoustic metagrating is designed for 2 MHz airborne ultrasound, since simulations using only the Helmholtz equation become inaccurate. However, it demonstrates also that metagratings can be designed to be still effective under pronounced thermoviscous effects.

**Fig. 1:**
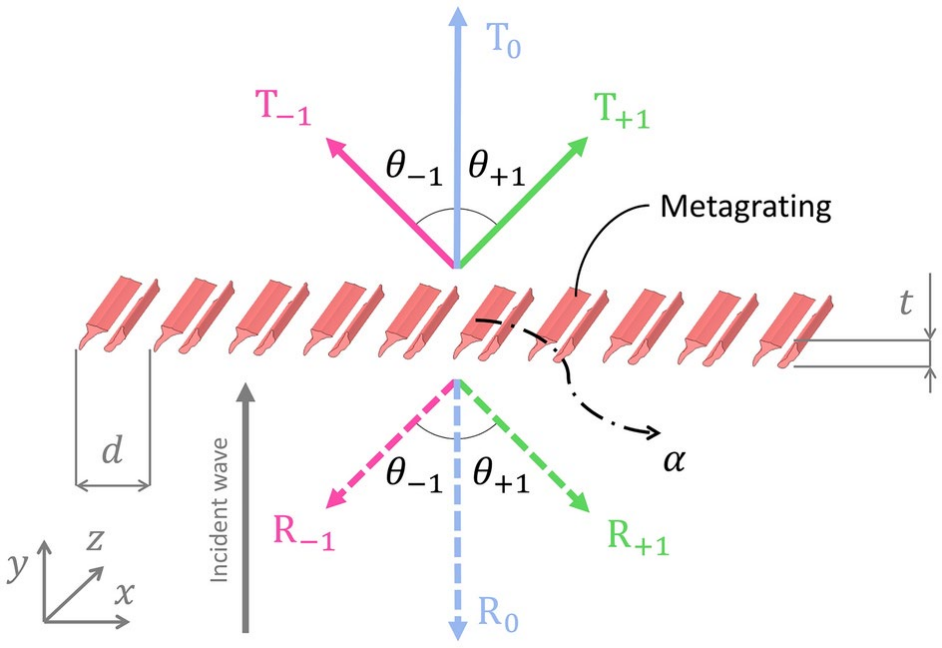
Microacoustic metagrating with three diffraction orders and absorption $\alpha$ under a normally incident wave. The diffraction order is indicated by the subscripts and refracted and reflected wave propagation directions are indicated by solid and dashed arrows, respectively. The grating parameters are the lattice constant *d* and the grating thickness *t*. (Adopted from Ref.^38^).

Acoustic metagratings can be considered as a subclass of the more general class of acoustic metamaterials and are periodic arrays of discrete meta-atoms capable of diffracting incident acoustic energy into multiple diffraction orders (cf. diffraction grating), as depicted in Fig. 1. The number of modes *n* and their corresponding diffraction angles $\theta _n$ are determined by the acoustic wavelength $\lambda$ and the metagrating lattice constant *d*, following Bragg’s condition: $d = n \lambda / \sin {\theta _n}$. Furthermore, the shape of the meta-atom is designed in such a way that the incident wave is transmitted towards one or more diffraction orders, while unwanted orders are fully cancelled. Experimental demonstrations of acoustic metagratings have been conducted usually in the audible frequency range: e.g. Craig et al. showcased a design at 6 kHz^33^, Hou et al. achieved operation at 8.2 kHz^32^ and Chiang et al. in the range from 2.43 to 2.54 kHz^34^. Notably, Chiang et al. also demonstrated the scalability of reflecting acoustic metagratings to the low-frequency ultrasound range with a design at 40 kHz^36^.

Microacoustic metagratings consist of micro-fabricated structures suitable for very high frequencies, and their small scale makes them highly susceptible to the influence of thermoviscous effects^38^. The contribution of viscosity becomes significant when the thickness of the Stokes’ boundary layer, given by $\delta _{\textrm{S}} = 2 \pi \sqrt{2 \mu / (\omega \rho _0)}$, approaches the dimensions of the channels^28,29^. Here, $\mu$ represents dynamic viscosity, $\rho _0$ is the equilibrium density, and $\omega = 2 \pi f$ denotes the angular frequency. We introduce a wavelength-normalized Stoke’s boundary layer thickness $\beta _{\textrm{S}} = \frac{\delta _S}{\lambda } = \frac{ \sqrt{f}}{c_0 }\sqrt{\frac{4 \pi \mu }{\rho _0}}$^28,29,38^. Compared to gradient metasurfaces, metagratings can have much coarser geometric features, allowing the boundary layer thicknesses to remain smaller in comparison, when shifting to the range of ultra-high frequency airborne ultrasound as shown in Fig. 1b of Ref.^38^. Recently, the first successful experimental demonstration of anomalous refraction with microacoustic metagratings was reported for 2 MHz with $\beta _{\textrm{S}} \approx 10^{-1}$ for a diffraction angle of –35 °^38^. However, in this work the key geometric features of the meta-atom were designed by parametric optimization (Design A in Ref.^38^) and topology optimization (initial geometry for shape optimization of Design C in Ref.^38^) without consideration of thermoviscous effects. Subsequently, only a small final reshaping was added by considering the thermoviscous effects within a shape optimization, while the evolution of the geometrical key features was driven without considering thermoviscous effects. This issue is driven by challenges in implementation of a stable numerical model using linearized Navier–Stokes equations on a strongly deformed mesh. Subsequently it is of great interest to implement an optimization without this limitation and to generate meta-atoms shapes which considers thermoviscous effects from the very beginning of geometry evolution. This would be a more natural way, closer to the evolution in nature, where all physical effects are active all the time and therefore one could expect geometries with better efficacy.

In this work, we present two shape-optimized geometries of microacoustic metagratings operating in the realm of airborne ultra-high frequency ultrasound, which offers a pathway to utilize acoustic metamaterials for applications such as flat acoustics, ultrasound imaging, and acoustic spectroscopy with enhanced spatial and temporal resolution. The optimization target is chosen to be the same as in Ref.^38^: refraction of a normally incident acoustic wave towards –35 ° at 2 MHz in air. The grating constant is set as *d* = 299 μm and therefore only three propagating diffraction orders (–1, 0, and +1) for both refracted and reflected waves are supported. Consequently, the metagrating enables redirection of the wave in six different directions, comprising three transmitted ($T_{-1}$, $T_{0}$, and $T_{+1}$) and three reflected ($R_{-1}$, $R_{0}$, and $R_{+1}$) diffraction orders, see Fig. 1. Additionally, due to significant contribution of the thermoviscous effects at $\beta _{\textrm{S}} \approx 10^{-1}$ a considerable part of the wave is absorbed creating a seventh degree of freedom $\alpha$. To design the meta-atoms for microacoustic metagratings in this work, we use the linearized Navier–Stokes equations discretized by the finite element method (FEM) as provided by Thermoviscous Acoustics physics of COMSOL Acoustic Module. The geometries are generated through FEM-based shape optimization to maximize the energy transmitted into the $-1$st diffraction order. We incorporate thermoviscous effects into the optimization process from the beginning, to avoid the optimizer converging towards a solution that may no longer be globally optimal once thermoviscous losses are considered. The neutral choice of the initial geometry ensures that the development of the geometrical key features is influenced by the presence of thermoviscous effects. This approach differs from the optimization strategy presented in Ref.^38^, where the optimization was performed in two steps. In the first step of Ref.^38^, the Helmholtz equation was used to determine an optimal geometry without considering thermoviscous losses. In the second step, this geometry was slightly adjusted to improve performance, taking thermoviscous losses into account. We demonstrate that considering thermoviscous effects from the beginning of the optimization leads to improved performance for anomalous refraction. Subsequently, we fabricate two different microacoustic metagratings using the two-photon polymerization technique. We experimentally validate the designed metagratings and compare the outcomes with the results presented in Ref.^38^.

## Results

### Evolution of optimized meta-atoms

**Fig. 2:**
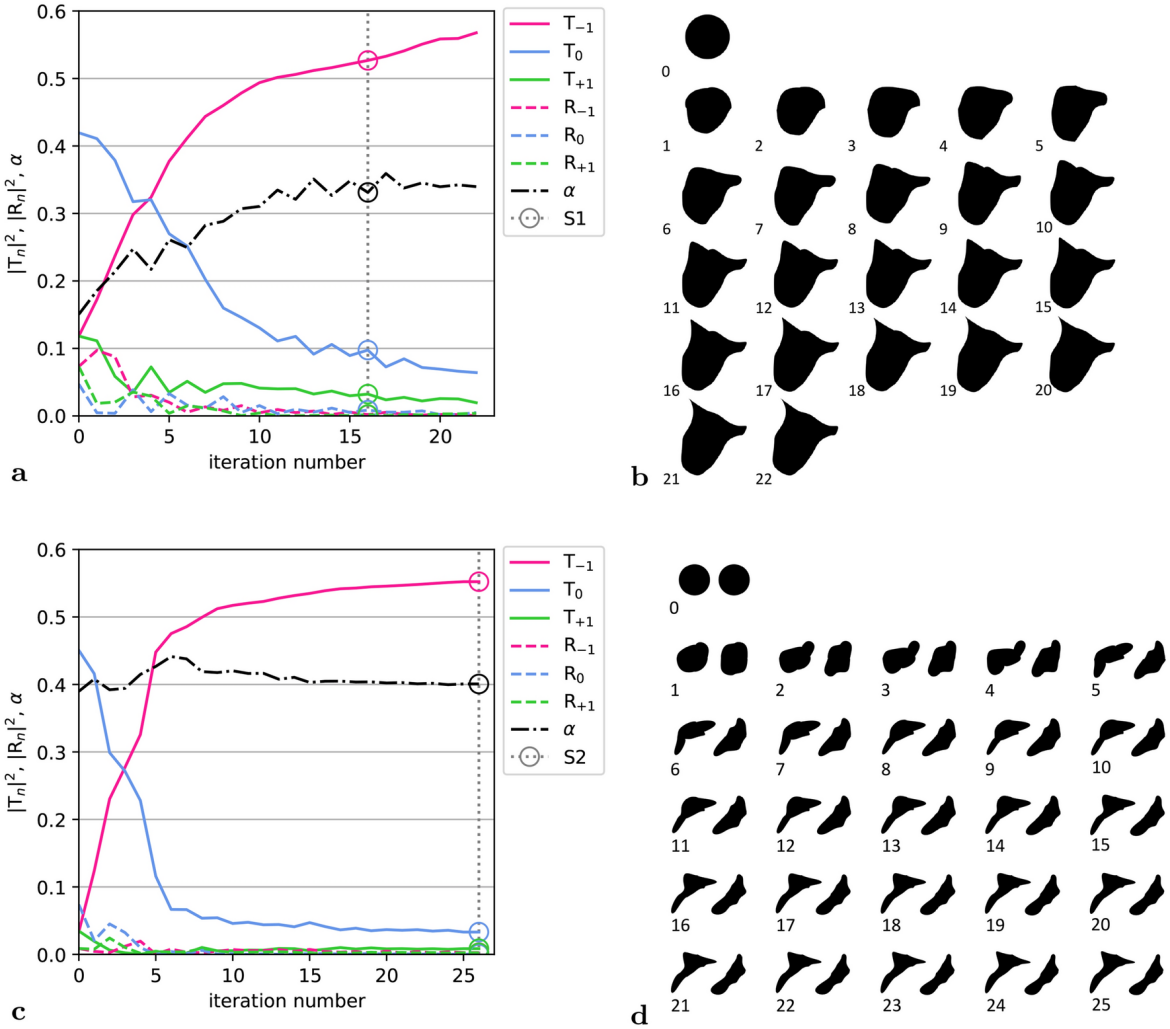
(**a**) Transmission, reflection and absorption during the optimisation process of S1. (**b**) Evolution of Shape 1 (S1) within 22 iterations. (**c**) Transmission , reflection and absorption during the optimisation process of S2. (**d**) Evolution of shape 2 (S2) within 25 iterations. (Optimization was implemented using COMSOL multiphysics optimization module).

In the following two different meta-atoms were designed using optimization resulting in a single-body (S1) and a two-body (S2) geometry. The shape optimization was conducted using COMSOL Multiphysics Optimization Module in order to maximize the transmission towards -1st diffraction order under consideration of thermoviscous effects. In both cases the initial geometry was deformed based on Bernstein polynomials of order 10 with a deformation limit of $\lambda /2$ throughout the complete optimization process. The evolution transmission, reflection and absorption coefficients, particularly of transmission $|T_{-1}|^2$ during the optimization iterations of single-body and two-body initial geometries are shown in Fig. 2a and c, respectively. In both cases it was possible to achieve a performance $|T_{-1}|^2 > 0.5$.

For the single-body meta-atom (S1) the initial geometry was a single circle with a radius of $0.4 \lambda$. The geometry evolution during the iteration is shown in Fig. 2b. After 6 iterations a transmission $|T_{-1}|^2 > 0.4$ is already reached, while the lengthy arm on the right is presumably the key feature of the final geometry, see Discussion section. After 16 iterations the optimizer starts to create a sharp corner on the top-left of the meta-atom. Since such sharp feature could cause problems for the manufacturing, iteration 16 was chosen for the final geometry despite the fact that better performance is predicted numerically at higher iteration numbers (see Fig. 2a). A meta-grating consisting of S1 geometry has a thickness of 199 μm or $1.16 \lambda$.

The two-body meta-atom is inspired by Design C of Ref.^38^, where it combines the positive aspects of a discrete meta-grating and of a gradient metasurface. The two channels can create a difference in the phase shift like in a gradient metasurface, while increasing the overall performance of anomalous refraction. For the two-body meta-atom (S2) the initial geometry defined by two circles with a radius of $\lambda /4$ and a distance of $0.58 \lambda$ between the centers aligned at equal *y* coordinate (refer to Fig. 1). The geometric evolution during the iteration is shown in Fig. 2d. In this case maximizing $|T_{-1}|$ requires fewer steps and after 5 iterations we reach a transmission of $|T_{-1}|^2 > 0.4$.

In the initial geometry (see Fig. 2d, iteration 0) (cf. Fig. 4a), two channels of different width are present. During the first 5 iterations the shape and length of the channels evolve to create strong transmission $T_{-1}$, and the key geometric features of the final design have already emerged. During the subsequent iterations the increase of $T_{-1}$ is relatively small between each step, while the localized geometrical features still continue to evolve. Nevertheless, the last iteration 25 is chosen for manufacturing, since it has no geometric features that are difficult to fabricate, while performing the best. The thickness of the S2 meta-grating is 111 μm$\approx 0.65 \lambda$ and smaller than the thickness of S1 and additionally of sub-wavelength dimension.

### Single-body meta-atom

**Fig. 3:**
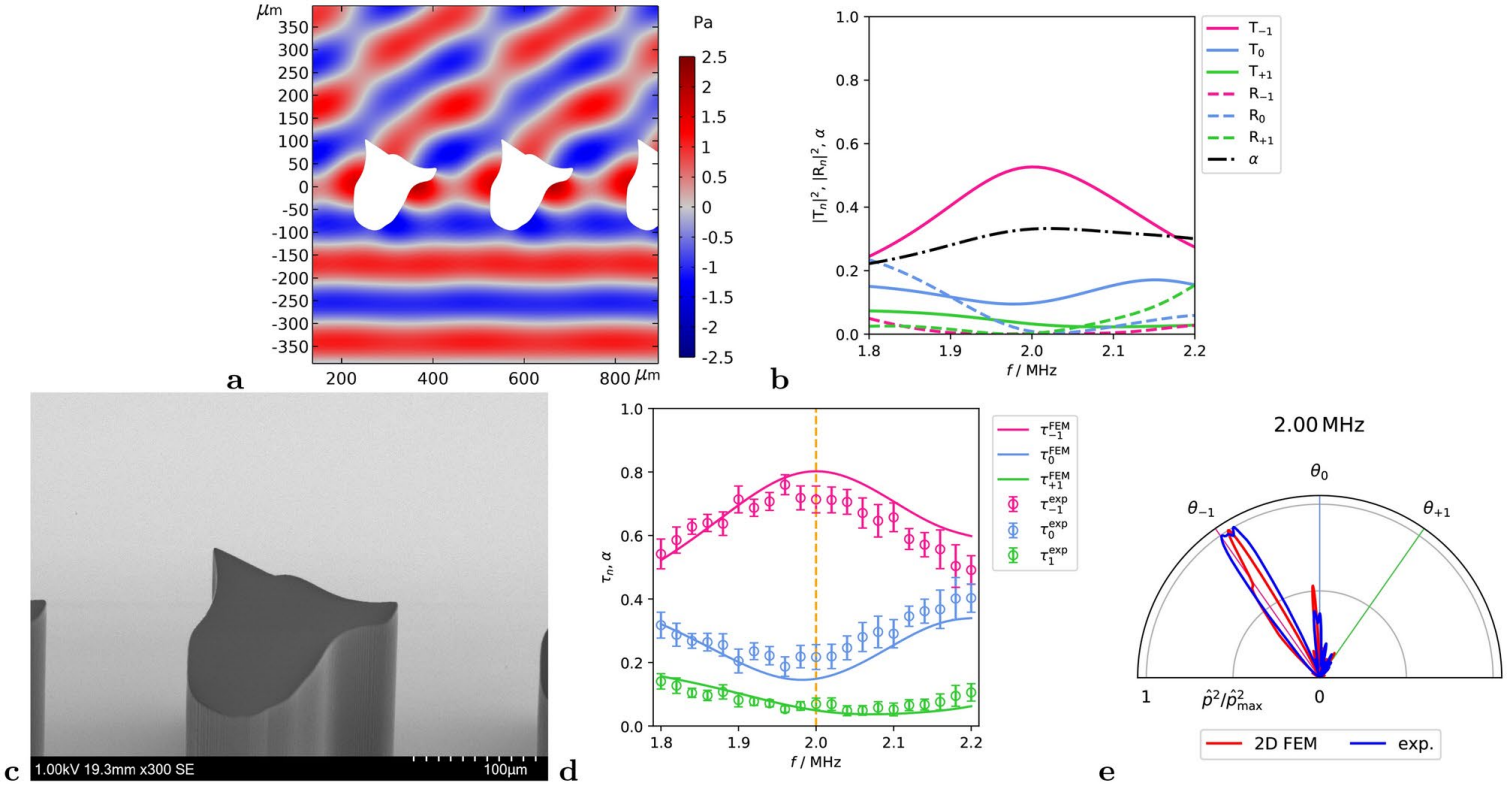
Numerical and experimental results for single-body meta-atom. (**a**) Numerically calculated pressure distribution at 2 MHz. (**b**) Numerically calculated transmission, reflection and absorption coefficients. (**c**) SEM micrograph of the manufactured meta-atom. (**d**) Numerically and experimentally determined normalized transmission. (**e**) Numerically (2D FEM with finite metagrating and ultrasonic outlet geometry) and experimentally determined directivity pattern at 2 MHz.

The simulated pressure distribution resulting from a normally incident wave hitting the final geometry is shown in Fig. 3a. The transmission at the target frequency 2 MHz is $|T_{-1}|^2 = 0.53$, see Fig. 3b, which actually is the maximum value in the investigated frequency range. At the same time we observe the absorption coefficient $\alpha$ over the entire analyzed frequency range staying below the maximum value of 0.33 at 2.02 MHz.

Figure 3c shows an SEM (scanning electron microscope) image of the manufactured metagrating sample. The measured and simulated transmission are normalized as per Eq. (9), and are shown in Fig. 3d, where both results match each other. The measurement setup and the experimental data analysis are described in Methods section, see *Experimental setup* and *Experimental data analysis*. From the numerics we expect the $\tau _{-1}$ peak at 2.00 MHz with $\tau _{-1}=0.80$. In the experiment we observe the the $\tau _{-1}$ peak at 1.96 MHz with $\tau _{-1}=0.76 \pm 0.06$, which is 5 % below the simulated peak value. In Fig. 3e the measured and simulated directivity pattern at target frequency 2 MHz is shown, where we observe the energy concentrated in the lobe directed toward –35 ° with a slight deviation of the lobe towards smaller angles. This deviation is observed in experiment and simulation and is linked to the finite number of the meta-atoms in the metagrating.

### Two-body meta-atom

**Fig. 4:**
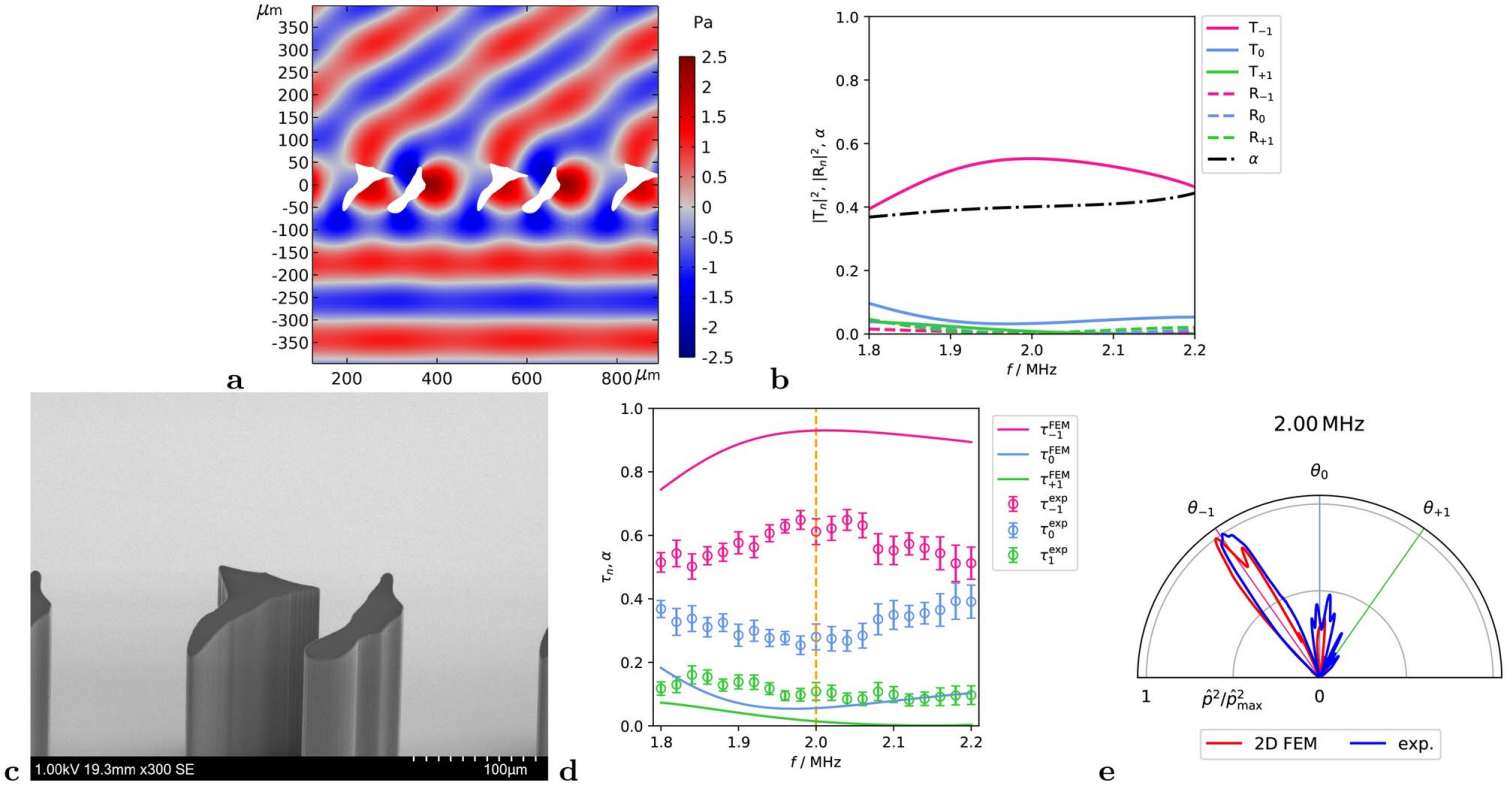
Numerical and experimental results for two-body meta-atom. (**a**) Numerically calculated pressure distribution at 2 MHz. (**b**) Numerically calculated transmission, reflection and absorption coefficients. (**c**) SEM micrograph of the manufactured meta-atom. (**d**) Numerically and experimentally determined normalized transmission. (**e**) Numerically (2D FEM with finite metagrating and ultrasonic outlet geometry) and experimentally determined directivity pattern at 2 MHz.

The resulting geometry and the pressure distribution from an incident wave at 2 MHz are shown in Fig. 4a. Figure 4b shows the numerically determined transmission, reflection, and absorption coefficients. This design offers a strong peak transmission with $|T_{-1}|^2 = 0.55$ at 2 MHz. In comparison to the single-body design, it demonstrates $|T_{-1}|^2 > 0.4$ over the entire investigated range, while the absorption coefficient $\alpha$ stays around 0.4. The increased $\alpha$ suggests that the losses within the S2 design are in general higher as in S1, which is the effect of narrower channels of S2 compared to S1. In addition, all other transmission and reflection coefficients lie below 0.1, which means that most of the energy goes to $T_{-1}$ or is absorbed.

An SEM image of the manufactured metagrating sample is shown in Fig. 4c. The experimentally determined and numerically calculated normalized energy distribution $\tau$ is shown in Fig. 4d. The measurement setup and the experimental data analysis are described in Methods section, see *Experimental setup* and *Experimental data analysis*. We observe that over 90 % of the energy is expected to be in the -1-lobe around the target frequency. The $\tau _{-1}$ maximum is located at 2.02 MHz with $\tau _{-1} = 0.93$. However, this result is not confirmed within the experiment, where a maximum is observed at 2.04 MHz with $\tau _{-1} = 0.65 \pm 0.03$, which is 30% below the expected value. Although there is energy leakage into the 0th and +1st diffraction modes, the –1st diffraction mode is dominant followed by 0th mode as it is also present in the numerical simulation.

In Fig. 4e the measured and simulated directivity pattern at target frequency 2 MHz is shown, where we observe the energy concentrated in the lobe directed toward −35 °, while some disturbed field is aligned around 0 °.

## Discussion

We note that the final shape of the S1 meta-atom has pronounced similarities to the Design B meta-atom reported in Ref.^38^, while the thickness of the meta-grating compared to Design B is slightly increased by 1.5 % to *t* = 199 μm. The prominent arm on the right side is also present in Design A and B in Ref.^38^, which suggests that this could be one of the general geometric key features for refraction towards –35 °. This right arm significantly contributes to the symmetry breaking of the geometry, and in Fig. 3a, a resonance below this arm can be observed. Previous works^39,40^ have shown that strong local resonances are critical to achieving maximal Willis coupling, which cancels out the forward-directed scattered field and redirects the wave in the desired direction. In addition, the pressure field in Fig. 3a is very close to the pressure field in Supplementary Fig. S1a of Ref.^38^. When comparing the experimental results, the peak performance of the S1 metagrating with a maximum of $\tau _{-1}=0.76 \pm 0.06$ at 1.96 MHz is slightly lower then the performance of the Design B from Ref.^38^ with a maximum of $\tau _{-1}=0.83 \pm 0.03$ at 2.02 MHz. At the same time S1 performs better than Design B at frequencies away from the designed frequency.

Since these two completely different optimization strategies delivered similar geometry types, it can be expected that both geometries are close to the optimal geometry for best –35 ° refraction performance at normalized Stokes boundary layer thickness around 0.081. Furthermore we note that the results of the S1 geometry in this work as well as the performance of Design A from Ref.^38^ are both supported by the experimental validation within the same experimental setup.

In case of the S2 geometry, the numerical results suggest significantly improved performance with better broadband properties and a maximum of $\tau _{-1} = 0.93$ (Fig. 3b) versus $\tau _{-1} = 0.90$ of Design C from Ref.^38^. Considering numerical calculations only leads to a conclusion that the improved optimization strategy results in better performing geometries. Unfortunately, it was not possible to confirm the promising numerical results in experiment while discrepancies in the spectra are present in Fig. 3d. Since also the experimental evaluation of S2 metagrating has been conducted at the same setup as the experiments in Ref.^38^, it is unlikely an issue of the experimental setup. However, in the directivity pattern additional peaks are present not following the diffraction angles given by Bragg’s condition, which is a sign of disturbed periodicity likely to be caused by unwanted deformation. With our equipment it is not possible to track if any unwanted deformation of the beams occurs in the mounted state, while unwanted deformation cannot be excluded. During the handling the microscopic beams can also break, while it actually happened to one of the pre-samples with S2 geometry. To prevent experimental evaluation on broken samples the actually measured sample was inspected on damage before and after the measurement. Due to transparency the the IP-S photoresist and limited view it is not guaranteed that every kind of damage or deflection can be found by microscopy, however no damage of the measured sample could be identified. Furthermore, the acoustic transmission of air at ultra high frequencies was not measured during the experiments. It is not known how sensitive the designs are to deviations in material parameters.

When comparing the experimental results of S2 metagrating with a maximum of $\tau _{-1} = 0.65 \pm 0.03$ at 2.04 MHz vs. S1 metagrating with a maximum of $\tau _{-1}=0.76 \pm 0.06$, S1 is outperforming S2 considering $\tau _{-1}$, while S2 is still beneficial from the thickness point of view. However, when comparing the transmission performance of the two-body geometry called Design C in Ref.^38^ with the performance of S2 metagrating with a maximum of $\tau _{-1} = 0.90 \pm 0.02$ at 1.96 MHz, it is clear that Design C by far outperforms the S2 geometry considering the current experiment. However, from the numerical consideration S2 is comparable with Design C from Ref.^38^, while S2 is even outperforming Design C around 2.2 MHz. We note that thermoviscous losses present an ultimate limit on the efficiency, since they dissipate a certain proportion of incident energy, as can be calculated e.g. by Stinson’s formulas^41^. However, further work is needed to reach this optimum, since we still have significant diffraction and reflection into unwanted order.

As a final remark it has to mentioned that according to the boundary layer thickness the herein presented results for airborne ultrasound can be transferred to waterborne ultrasound using the wave-length normalized boundary layer thicknesses, see Ref.^38^. This assumption is not perfectly accurate, since the Prandtl numbers of air and water are different, $\textrm{Pr}^{\textrm{water}} = 7.1$ vs. $\textrm{Pr}^{\textrm{air}} = 0.71$. When neglecting the thermal boundary layer effects, a transfer based on the normalized Stokes boundary layer thickness would lead to a waterborne study at 559 MHz with a grating constant of *d* = 4.63 μm.

## Conclusions

We demonstrated two different metagrating designs, based on a single-body meta-atom (S1) and on a two-body meta-atom (S2), while the geometries were found by a shape-optimization approach including thermoviscous effects. The chosen modelling and optimization technique allows to include thermoviscous effects during the complete optimization routine. Both designs were manufactured and proven experimentally, while in both cases anomalous refraction towards –35 ° at 2 MHz was demonstrated. In the case of S1 meta-atom the geometric key features of recently reported geometries in Ref.^38^ could be confirmed, while the overall performance is comparable to Ref.^38^. In the case of S2 meta-atom the numerically calculated performance was significantly better compared to the recently published two-body design (Design C from Ref.^38^). During the experiment the general function of S2 was confirmed, however the measured performance was lower compared to the model and compared to literature^38^. The experimental setup could be developed to further reduce its sensitivity to manufacturing and assembling tolerances, as well as to potential deformation of the sample due to stresses from mounting. These results help to push forward the acoustic metamaterials and metagratings for microacoustic regimes, as it is the case for ultra-high frequency ultrasound or sound propagation in viscous fluids.

## Methods

### Numerical model

The COMSOL Multiphysics software was used to solve the linearized Navier–Stokes model for numerical solution. The model assumed an infinite metagrating in two-dimensional space, but only a single strip of length *d* was actually modeled. To maintain the periodicity of the solution, a Bloch-Floquet boundary condition was applied. Perfectly matched layers (PML) were used to model the infinite domain extension above and below the metagrating. This model was used for optimization and for calculating the spectra in Figs. 3b-d and 4b-d.

To calculate the plane wave expansion coefficients, a surface *A* with dimensions $d \times d$ was defined both before (reflection, *R*) and after (transmission, *T*) the metagrating leading to

1 $$\begin{aligned} \begin{aligned} a_n^T&= \frac{1}{A_T} \int _{A_T} p(\textbf{r}, f) e^{i \textbf{k}_n^T \textbf{r}} \text {d}A_T \\ a_n^R&= \frac{1}{A_R} \int _{A_R} p(\textbf{r}, f) e^{i \textbf{k}_n^R \textbf{r}} \text {d}A_R \\ a_{\textrm{inc}}&= \frac{1}{A_R} \int _{A_R} p(\textbf{r}, f) e^{i \textbf{k}_{\textrm{inc}} \textbf{r}} \text {d}A_R \end{aligned} \end{aligned}$$

with $n \in \left\{ 0, \pm 1 \right\}$ being the diffraction order, $p({\textbf {r}}, f)$ being the complex valued pressure, and ${\textbf {r}}$ being the location vector. The wave vector ${\textbf {k}}$ is defined as follows

2 $$\begin{aligned} \begin{aligned} {\textbf {k}}_n^T&= {\textbf {e}}_y |k| \cos {\theta _n} + {\textbf {e}}_x |k| \sin {\theta _n} \\ {\textbf {k}}_n^R&= - {\textbf {e}}_y |k| \cos {\theta _n} + {\textbf {e}}_x |k| \sin {\theta _n} \\ {\textbf {k}}_{\textrm{inc}}&= {\textbf {e}}_y |k| \end{aligned} \end{aligned}$$

where $|k| = 2 \pi f / c_0$ and $\theta _n$ is the refraction angle. The transmission and the reflection coefficients are defined as

3 $$\begin{aligned} \begin{aligned}&T_n = \frac{ a^T_n }{ a_{\text {inc}} } \\&R_n = \frac{ a^R_n }{ a_{\text {inc}} } \end{aligned} \end{aligned}$$

and the absorption coefficient as

4 $$\begin{aligned} \alpha = 1 - \sum _{n} |T_n|^2 - \sum _{n} |R_n|^2 , \, n \in \{ 0, \pm 1 \}. \end{aligned}$$

The normalized transmission follows as^38^

5 $$\begin{aligned} \tau _n = \frac{|T_{n}|^2}{|T_{-1}|^2 + |T_{0}|^2 + |T_{+1}|^2}. \end{aligned}$$

To calculate the diffraction pattern in Figs. 3e and 4e, a large 2D FEM model was used. In this case, the linearized Navier–Stokes equations were applied only in the region of the metagrating, while the remaining domain was modeled using the Helmholtz equation. The geometry of the section located at the measurement plane was used, capturing the effects of the finite number of meta-atoms and the enclosed volume in the ultrasonic outlet. The setup was also assumed to be infinite in the z direction. For the extent of the modeled domain, refer to Fig. S2 in the supporting materials of Ref.^38^.

The material parameters used for air are equilibrium density $\rho _0 = 1.2\; {\hbox {kg}\; \hbox {m}^{-3}}$, speed of sound $c = 343\; {\hbox {m}\; \hbox {s}^{-1}}$, dynamic viscosity $\upmu = 1.814\;\times\; 10^{-5}]\; {\hbox {Pa}\;\hbox {s}}$, bulk viscosity $\upmu _{\textrm{B}} = 1.0884\;\times 10^{-5}\;{\hbox {Pa}\;\hbox {s}}$, thermal conductivity $k = 0.025768\;{\hbox {W}\; \hbox {m}^{-1}\; \hbox {K}^{-1}}$, heat capacity at constant pressure $C_p = 1005.4\;{\hbox {J} \;\hbox {kg}^{-1}\; \hbox {K}^{-1}}$, and ratio of specific heats $\gamma = 1.4$

### Sample manufacturing using two-photon lithography

The samples were fabricated using two-photon polymerization, which is a type of additive manufacturing. The fabrication process was carried out using the Photonic Professional GT2 from Nanoscribe GmbH, Germany. The build volume of the machine is $10\; \times\; 10 \times\; 0.8\; {\hbox {cm}^3}$^42^. The shape of the smallest unit of fabrication, known as a voxel, is elliptic. The size of the voxel, with dimensions ranging from 0.1 to 5 μm in the radial direction ($r_v$) and from 0.3 to 15 μm in the axial direction ($z_v$)^42^, depends on the parameters of the illumination process and the optical properties of the photoresist. To create the desired structures, the voxel is scanned through the photoresist based on positional data from CAD models. After the fabrication process, excess photoresist is removed, leaving behind the cured structures. These structures are then detached from the substrate after drying.

**Fig. 5:**
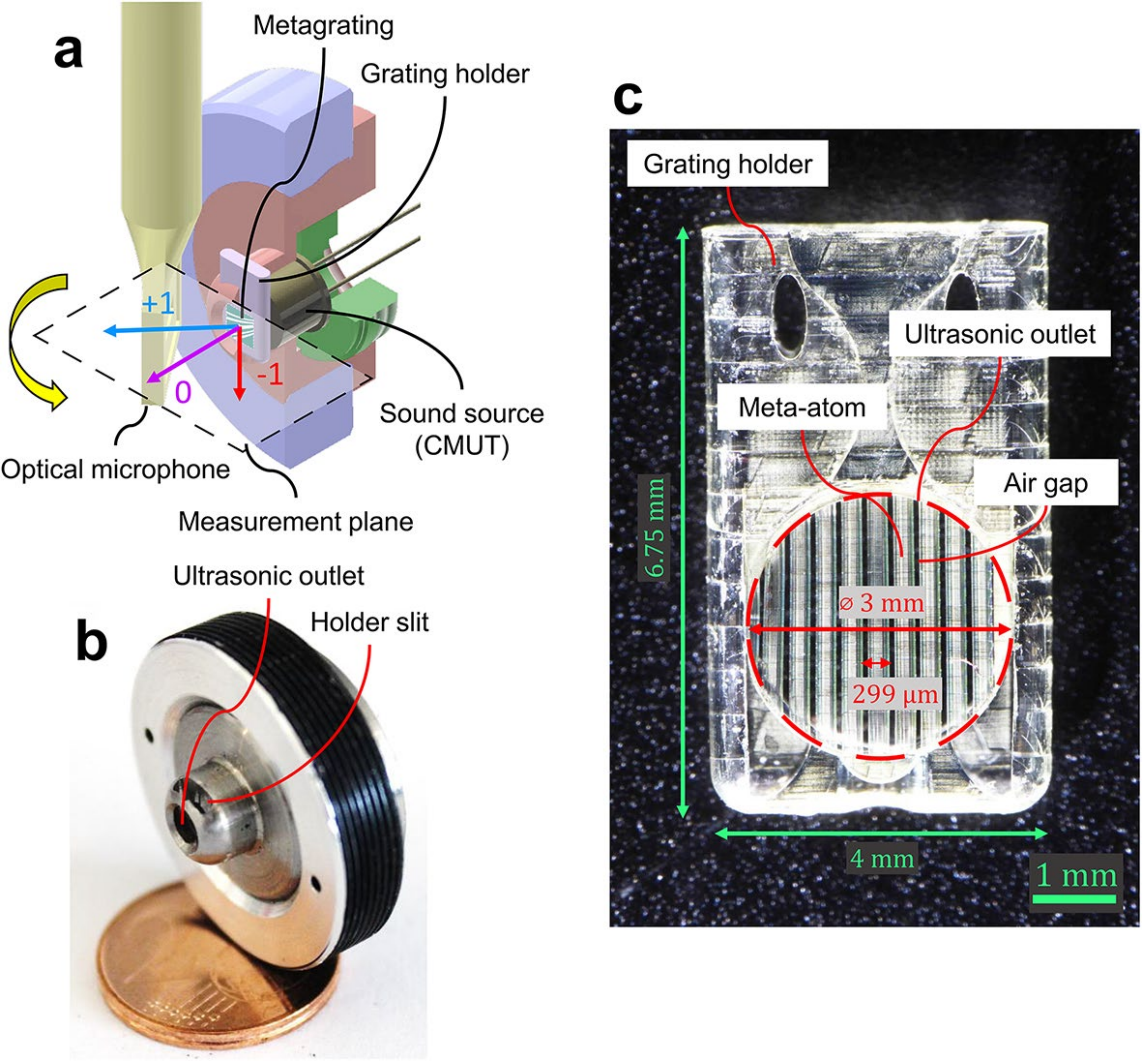
(**a**) Measurement setup consisting of the rotatable holder, the sound sorce (CMUT) and the moving laser microphone. (**b**) Real size photograph of the rotatable holder with ultrasonic outlet. (**c**) Microscope image of the metagrating in the grating holder. (a and b taken from Ref.^38^).

Focusing during the fabrication process was achieved using immersion objectives. A $10 \times$ objective with a numerical aperture (NA) of 0.3 was used for the grating holder (see Fig. 5c), while a $25 \times$ objective with a NA of 0.8 was used for the grating sample (see meta-atoms in Fig. 5c). The photoresists IP-Q and IP-S, provided by Nanoscribe GmbH, Germany, were used for the fabrication process. The development of the photoresist was carried out using 1-Methoxy-2-propylacetat for a duration of 20 min with a volume of 60 ml, followed by a 5-min development step using 2-propanol with a volume of 60 ml. After development, the samples were dried in air under a glass hood at a temperature of approximately $21.7 \pm 0.44$ degrees Celsius and a relative humidity of $32.1 \pm 8.2\%$. The resulting mechanical properties for the IP-S photoresist are as follows: a Poisson’s ratio of approximately 0.3, a Young’s modulus of approximately 5.11 GPa, and a density of approximately 1.22 g/cm^3^^42^.

### Experimental setup

The measurement setup utilized in this study is shown in Fig. 5a as schematic and in Fig. 5b in real size.^38^ It consists of an assembly that connects a sound source to a microacoustic metagrating. The metagrating supported by a grating holder (see Fig. 5c) can be manually placed into the holder slit (see Fig. 5b). The assembly is mounted on a position system, allowing for relative movement between the assembly and a microphone. The manufactured sample of the metagrating is finite in the x and z directions, while it completely fills the ultrasonic outlet area. This setup mimics a situation where the ultrasound field between the source and the metagrating is a plane wave. We note that the 2D approximation is justified by using a grating with a length in the z direction much longer than the acoustic wavelength and the incident beam width. Furthermore, we note that during the numerical optimization, an infinite metagrating in the x and z directions and an incident plane wave were assumed.

The sound source used in the setup is a capacitive micro-machined ultrasound transducer, which was designed and fabricated in-house^43^. The transducer is mounted on a TO-18 metal case and secured in the frame using a screw. To measure ultra-high frequency ultrasound, the Eta450 Ultra optical microphone from Xarion, Austria, is employed^44,45^. This microphone is specified to have a frequency range of 50 kHz to 2 MHz. However, in this study, the frequency range could be extended up to 2.2 MHz since only relative sound pressure values are utilized.

Motorized stages from Physical Instruments, Germany, are utilized for measuring radial scans in a single plane. The directional characteristics of the microphone are disregarded as it is aligned with the same side as the axis of rotation on the metagrating^46^. To avoid any collision between the rotatable holder and the microphone attachment, the measured angle is limited to $-70^{\circ }< \theta < +70^{\circ }$. To optimize measurement time, the radial axis is continuously moved, triggering sound pressure measurements at a resolution of 0.1 °. The measurements are conducted with continuous stimulation, employing a sinusoidal signal of 10 Vpp with a DC bias of 20 V. The metagrating is aligned with the rotation axis, which is perpendicular to the measurement plane, while the microphone’s center is positioned at half the height of the metagrating. The refracted wave is then measured using the optical microphone along a circular trajectory with a radius of 9.87 mm from the surface of the metagrating.

### Experimental data analysis

The raw measured pressure amplitude is fitted using a Gaussian mixture model of the form

5 $$\begin{aligned} G(\theta ) = \sum _{n} \hat{p}_{n} g_n(\theta ) \end{aligned}$$

where

6 $$\begin{aligned} g_n(\theta ) = e^{-\frac{(\theta - \theta _n)^2}{2 \sigma ^2}} \end{aligned}$$

with

7 $$\begin{aligned} n \in \{0, \pm 1\} \end{aligned}$$

Here, $\hat{p}_{n}$ is the pressure amplitude at the beam center and $\sigma$ corresponds to the beam width. The estimation of the pressure magnitude error is given by

8 $$\begin{aligned} \Delta \hat{p}_n = \sqrt{ \int _{\Theta } \left( p_{exp} \left( \theta \right) - \hat{p}_n g_n (\theta ) \right) ^2 w_n \left( \theta \right) \textrm{d}\Theta } \end{aligned}$$

where the weighting function is defined as $w_n \left( \theta \right) = g_n \left( \theta \right)$. The pressure amplitude $\hat{p}_{n}$ is utilized to calculate the normalized transmission within the diffraction orders as follows:

9 $$\begin{aligned} \tau ^{\text {exp}}_n = \frac{\hat{p}_{n}^2}{\sum _m \hat{p}_{m}^2},\,m \in \{ 0, \pm 1 \} \end{aligned}$$

This calculation is connected to Eq. () which represents the equation for the normalized transmission. The introduction of this quantity is necessary due to the challenges associated with measuring absolute power and considering multiple reflections. The propagation of the error is subsequently determined using the equation:

10 $$\begin{aligned} \Delta \tau ^{\text {exp}}_{i} = 2 \sqrt{\frac{\hat{p}_{i}^4 \left( \Delta \hat{p}_j^2 \hat{p}_j^2 + \Delta \hat{p}_{k}^2 \hat{p}_{k}^2 \right) + \Delta \hat{p}_{i}^2 \hat{p}_{i}^2 \left( \hat{p}_j^2 + \hat{p}_{k}^2 \right) ^2 }{\left( \hat{p}_{i}^2 + \hat{p}_{j}^2 + \hat{p}_{k}^2\right) ^4}} \end{aligned}$$

where $i \in \{ -1, 0, +1 \}$, $j \in \{ 0, +1, -1 \}$, and $k \in \{ +1, -1, 0 \}$. This calculation leads to the error bars shown in Figs. 3d and 4d.

## Data Availability

The datasets used and/or analysed during the current study available from the corresponding author on reasonable request.

## References

[1] Cummer, S. A., Christensen, J. & Alù, A. Controlling sound with acoustic metamaterials. *Nat. Rev. Mater.Bold">1*, 1–13 (2016).

[2] Haberman, M. R. & Guild, M. D. Acoustic metamaterials. *Phys. Today* **69**, 42–48 (2016).

[3] Ma, G. & Sheng, P. Acoustic metamaterials: From local resonances to broad horizons. *Sci. Adv.* **2**, e1501595 (2016). 26933692 10.1126/sciadv.1501595 PMC4771441

[4] Jiménez, N. et al. (eds) *Acoustic waves in periodic structures, metamaterials, and porous media: From fundamentals to industrial applications* Vol. 143 (Springer, Cham, 2021).

[5] Farhat, M., Guenneau, S. & Enoch, S. Ultrabroadband elastic cloaking in thin plates. *Phys. Rev. Lett.* **103**, 024301 (2009). 19659209 10.1103/PhysRevLett.103.024301

[6] Sanchis, L. et al. Three-dimensional axisymmetric cloak based on the cancellation of acoustic scattering from a sphere. *Phys. Rev. Lett.* **110**, 124301 (2013). 25166808 10.1103/PhysRevLett.110.124301

[7] Zigoneanu, L., Popa, B.-I. & Cummer, S. A. Three-dimensional broadband omnidirectional acoustic ground cloak. *Nat. Mater.* **13**, 352–355 (2014). 24608143 10.1038/nmat3901

[8] Cheng, Y. et al. Ultra-sparse metasurface for high reflection of low-frequency sound based on artificial Mie resonances. *Nat. Mater.* **14**, 1013–1019 (2015). 26322718 10.1038/nmat4393

[9] Wu, X. et al. High-efficiency ventilated metamaterial absorber at low frequency. *Appl. Phys. Lett.Bold">112*, 103505 (2018).

[10] Melnikov, A. et al. Acoustic metamaterial capsule for reduction of stage machinery noise. *J. Acoust. Soc. Am.* **147**, 1491. 10.1121/10.0000857 (2020). 32237831 10.1121/10.0000857

[11] Kaina, N., Lemoult, F., Fink, M. & Lerosey, G. Negative refractive index and acoustic superlens from multiple scattering in single negative metamaterials. *Nature* **525**, 77–81 (2015). 26333466 10.1038/nature14678

[12] Chen, J., Xiao, J., Lisevych, D., Shakouri, A. & Fan, Z. Deep-subwavelength control of acoustic waves in an ultra-compact metasurface lens. *Nature Commun.* **9**, 4920 (2018). 30467347 10.1038/s41467-018-07315-6 PMC6250707

[13] Jin, Y., Kumar, R., Poncelet, O., Mondain-Monval, O. & Brunet, T. Flat acoustics with soft gradient-index metasurfaces. *Nat. Commun.* **10**, 143 (2019). 30635556 10.1038/s41467-018-07990-5 PMC6329837

[14] Xia, J. et al. Acoustofluidic virus isolation via bessel beam excitation separation technology. *ACS Nano* **18**, 22596–22607. 10.1021/acsnano.4c09692 (2024). 39132820 10.1021/acsnano.4c09692 PMC12212744

[15] Li, J., Fok, L., Yin, X., Bartal, G. & Zhang, X. Experimental demonstration of an acoustic magnifying hyperlens. *Nat. Mater.* **8**, 931–934 (2009). 19855382 10.1038/nmat2561

[16] Zhu, J. et al. A holey-structured metamaterial for acoustic deep-subwavelength imaging. *Nat. Phys.* **7**, 52–55 (2011).

[17] Cheng, Y., Zhou, C., Wei, Q., Wu, D. & Liu, X. Acoustic subwavelength imaging of subsurface objects with acoustic resonant metalens. *Appl. Phys. Lett.Bold">103*, 224104 (2013).

[18] Li, Y. et al. Experimental realization of full control of reflected waves with subwavelength acoustic metasurfaces. *Phys. Rev. Appl.* **2**, 064002 (2014).

[19] Guo, J., Zhang, X. & Fang, Y. Topology optimization design and experimental validation of an acoustic metasurface for reflected wavefront modulation. *J. Sound Vib.* **520**, 116631 (2021).

[20] Xie, Y. et al. Wavefront modulation and subwavelength diffractive acoustics with an acoustic metasurface. *Nat. Commun.* **5**, 5553 (2014). 25418084 10.1038/ncomms6553

[21] Molerón, M., Serra-Garcia, M. & Daraio, C. Acoustic Fresnel lenses with extraordinary transmission. *Appl. Phys. Lett.* **105**, 114109. 10.1063/1.4896276 (2014).

[22] Liu, B. et al. Experimental realization of all-angle negative refraction in acoustic gradient metasurface. *Appl. Phys. Lett.* **111**, 221602. 10.1063/1.5004005 (2017).

[23] Lan, J., Li, Y. & Liu, X. Broadband manipulation of refracted wavefronts by gradient acoustic metasurface with V-shape structure. *Appl. Phys. Lett.* **111**, 263501 (2017).

[24] Lan, J., Li, Y., Xu, Y. & Liu, X. Manipulation of acoustic wavefront by gradient metasurface based on Helmholtz resonators. *Sci. Rep.* **7**, 10587 (2017). 28878264 10.1038/s41598-017-10781-5 PMC5587551

[25] Li, J., Shen, C., Díaz-Rubio, A., Tretyakov, S. A. & Cummer, S. A. Systematic design and experimental demonstration of bianisotropic metasurfaces for scattering-free manipulation of acoustic wavefronts. *Nat. Commun.* **9**, 1342 (2018). 29632385 10.1038/s41467-018-03778-9 PMC5890261

[26] Zhang, T.-C. et al. Observation of ultrabroadband acoustic focusing based on V-shaped meta-atoms. *Adv. Mater. Technol.* **5**, 1900956 (2020).

[27] He, J. et al. Broadband three-dimensional focusing for an ultrasound scalpel at megahertz frequencies. *Phys. Rev. Appl.* **16**, 024006 (2021).

[28] Ward, G. P. et al. Boundary-layer effects on acoustic transmission through narrow slit cavities. *Phys. Rev. Lett.* **115**, 044302 (2015). 26252688 10.1103/PhysRevLett.115.044302

[29] Cotterill, P. A., Nigro, D., Abrahams, I. D., Garcia-Neefjes, E. & Parnell, W. J. Thermo-viscous damping of acoustic waves in narrow channels: A comparison of effects in air and water. *J. Acoust. Soc. Am.* **144**, 3421 (2018). 30599677 10.1121/1.5078528

[30] Torrent, D. Acoustic anomalous reflectors based on diffraction grating engineering. *Phys. Rev. B* **98**, 060101 (2018).

[31] Ni, H., Fang, X., Hou, Z., Li, Y. & Assouar, B. High-efficiency anomalous splitter by acoustic meta-grating. *Phys. Rev. B* **100**, 104104 (2019).

[32] Hou, Z., Fang, X., Li, Y. & Assouar, B. Highly efficient acoustic metagrating with strongly coupled surface grooves. *Phys. Rev. Appl.* **12**, 034021 (2019).

[33] Craig, S. R., Su, X., Norris, A. & Shi, C. Experimental realization of acoustic bianisotropic gratings. *Phys. Rev. Appl.* **11**, 061002 (2019).

[34] Chiang, Y. K. et al. Reconfigurable acoustic metagrating for high-efficiency anomalous reflection. *Phys. Rev. Appl.* **13**, 064067 (2020).

[35] Qian, J. et al. Aperiodic metagratings for high-performance multifunctional acoustic lenses. *Adv. Mater. Technol.* **5**, 2000542 (2020).

[36] Chiang, Y. K. et al. Scalable metagrating for efficient ultrasonic focusing. *Phys. Rev. Appl.* **16**, 064014 (2021).

[37] Song, A., Sun, C., Bai, Y., Xiang, Y. & Xuan, F.-Z. Reconfigurable acoustic metagrating for multiple anomalous wavefront manipulation functionalities. *Phys. Lett. A* **453**, 128477 (2022).

[38] Melnikov, A. et al. Microacoustic metagratings at ultra-high frequencies fabricated by two-photon lithography. *Adv. Sci.* **9**, 2200990 (2022). 10.1002/advs.202200990 PMC9284164 35466579

[39] Quan, L., Ra’di, Y., Sounas, D. L. & Alù, A. Maximum Willis coupling in acoustic scatterers. *Phys. Rev. Lett.* **120**, 254301 (2018). 29979059 10.1103/PhysRevLett.120.254301

[40] Melnikov, A. et al. Acoustic meta-atom with experimentally verified maximum Willis coupling. *Nat. Commun.* **10**, 1–7 (2019). 31316062 10.1038/s41467-019-10915-5 PMC6637156

[41] Stinson, M. R. The propagation of plane sound waves in narrow and wide circular tubes, and generalization to uniform tubes of arbitrary cross-sectional shape. *J. Acoust. Soc. Am.* **89**, 550–558. 10.1121/1.400379 (1991).

[42] NanoGuide. NanoGuide. https://support.nanoscribe.com/hc/en-gb/articles/360001748953-IP-Dip (2021).

[43] Koch, S. G. et al. Empowering robots for multimodal tactile gripping using capacitive micromachined ultrasonic transducers. In *2021 smart systems integration (SSI)* 1–5 (IEEE, Piscataway, 2021).

[44] Fischer, B. Optical microphone hears ultrasound. *Nat. Photonics* **10**, 356–358 (2016).

[45] XARION Laser Acoustics GmbH. Eta450 ultra: Datasheet.

[46] Preisser, S. et al. All-optical highly sensitive akinetic sensor for ultrasound detection and photoacoustic imaging. *Biomed. Opt. Express* **7**, 4171–4186 (2016). 27867723 10.1364/BOE.7.004171 PMC5102516

